# Transcriptome Characterization of Reverse Development in *Turritopsis dohrnii* (Hydrozoa, Cnidaria)

**DOI:** 10.1534/g3.119.400487

**Published:** 2019-10-16

**Authors:** Yui Matsumoto, Stefano Piraino, Maria Pia Miglietta

**Affiliations:** * Texas A&M University at Galveston, Galveston, TX and; † Università del Salento, Dipartimento di Scienze e Tecnologie Biologiche ed Ambientali, 73100 Lecce, Italy

**Keywords:** immortal jellyfish, reverse metamorphosis, life-cycle reversal, cell transdifferentiation, RNA-sequencing

## Abstract

Medusae of *Turritopsis dohrnii* undergo reverse development in response to physical damage, adverse environmental conditions, or aging. Senescent, weakened or damaged medusae transform into a cluster of poorly differentiated cells (known as the cyst stage), which metamorphose back into a preceding life cycle stage, the polyp. During the metamorphosis, cell transdifferentiation occurs. The cyst represents the intermediate stage between a reverting medusa and a healthy polyp, during which cell transdifferentiation and tissue reorganization take place. Here we characterize and compare the transcriptomes of the polyp and newborn medusa stages of *T. dohrnii* with that of the cyst, to identify biological networks potentially involved in the reverse development and transdifferentiation processes. The polyp, medusa and cyst of *T. dohrnii* were sequenced through Illumina RNA-sequencing and assembled using a *de novo* approach, resulting in 92,569, 74,639 and 86,373 contigs, respectively. The transcriptomes were annotated and comparative analyses among the stages identified biological networks that were significantly over-and under-expressed in the cyst as compared to the polyp and medusa stages. Biological processes that occur at the cyst stage such as telomerase activity, regulation of transposable elements and DNA repair systems, and suppression of cell signaling pathways, mitotic cell division and cellular differentiation and development may be involved in *T. dohrnii*’s reverse development and transdifferentiation. Our results are the first attempt to understand *T. dohrnii*’s life-cycle reversal at the genetic level, and indicate possible avenues of future research on developmental strategies, cell transdifferentiation, and aging using *T. dohrnii* as a non-traditional *in vivo* system.

In a typical hydrozoan life cycle (Figure 1), adult, free-swimming medusae release gametes into the water column for external fertilization, producing short-living planktotrophic planula larvae with limited dispersal distance (Bouillon *et al.* 2006). The planula settles on the sea floor and metamorphoses into the colonial polyp stage, which will asexually propagate and bud new medusae, closing the life cycle. The hydrozoan *Turritopsis dohrnii* (Weismann 1883) (Filifera, Oceaniidae) exhibits an additional developmental trajectory: when physically damaged, senescent, or faced with adverse environmental conditions, the medusae of *T. dohrnii* avoid death by reversing their life-cycle in the opposite developmental direction, *i.e.*, transforming back into the post-larval, benthic polyp (Figure 1). During its reverse development, medusae shrink and lose their swimming competence, settle onto the substrate, and transform into a cyst-like stage characterized by a thin chitinous external envelope with no recognizable morphological features that can be ascribed to either medusa or polyp (Schmich *et al.* 2007). In the following 24-36 hr, the cyst develops polyp-typical features, such as the stolonal hydrorhiza, where new polyps will arise from, eventually returning to the customary polyp-to-medusa ontogenetic sequence (Martell *et al.* 2016). *Turritopsis dohrnii*’s reverse development is considered a metamorphosis (Müller and Leitz 2002; Berking 1998), albeit in the opposite direction of its normal developmental trajectory and has earned *T. dohrnii* the popular appellation of “immortal jellyfish”.

**Figure 1:**
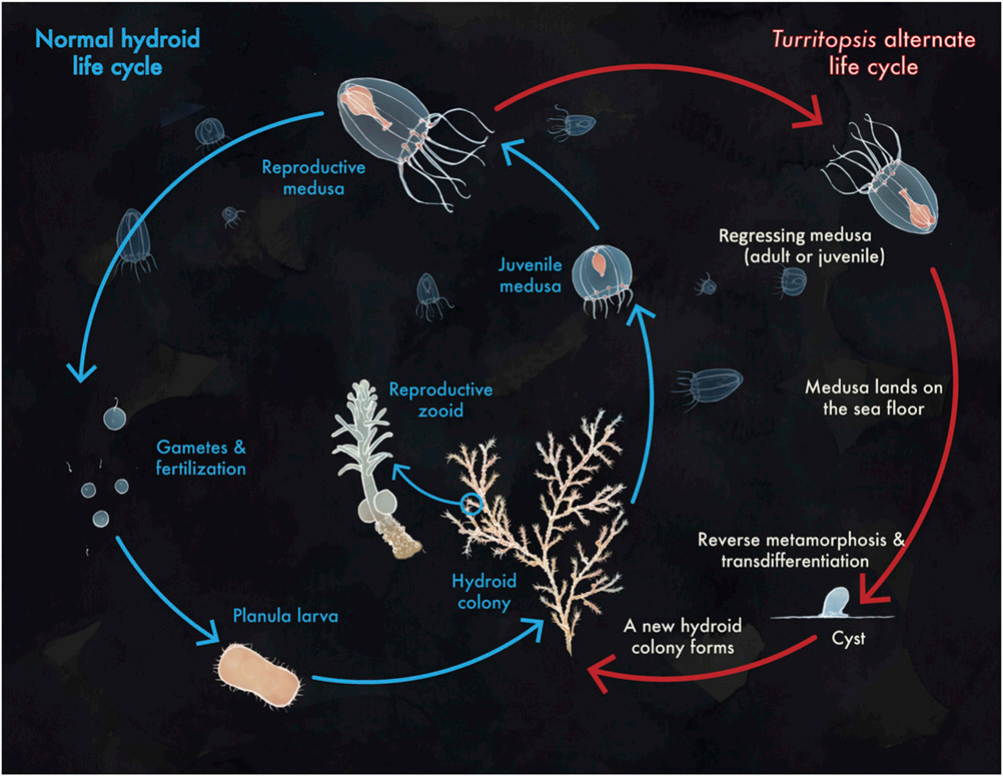
Life cycle of *Turritopsis dohrnii*, the Immortal Jellyfish. The typical hydrozoan life cycle is indicated in blue (left), while the alternate life cycle of *T. dohrnii* is indicated in red (right).

Polyps and medusae differ in anatomy and in cell types. Some cell types and sensory organs (*e.g.*, subumbrellar striated muscle cells, nerve rings, ocelli) are present only in the medusa, whereas other cell types (*e.g.*, ectodermal secretory cells producing the chitinous outer persiarc) occur in the polyps only. Tissue excision experiments have provided insight on the cellular mechanism that underlies life-cycle reversal in *T. dohrnii* (Piraino *et al.* 1996). The manubrium of the medusa contains a large population of interstitial cells (I-cells) that play a large role in cnidarian regeneration (Tardent 1963), while the exumbrellar epidermis does not contain any I-cell populations. When the two types of tissue were excised and isolated, the manubrium (containing I-cells) was not able to transform into perisarc-secreting tissue, the chitinous exterior that protects the soft tissue in the cyst and polyp stage, but absent in the medusa (Piraino *et al.* 1996). The exumbrellar epidermis (without I-cells) however, transformed into perisarc-secreting tissue, and was able to reverse develop into a juvenile polyp. Thus, although I-cell proliferation occurs during the reversal, it does not seem to play a crucial role in the morph rejuvenation from medusae to polyp as portions of the medusae that contain no I-cells are still able to fully revert to the polyp stage (Piraino *et al.* 1996). Given the limited role of I-cells and the fact that medusae and polyps contain different cell types, the transformation from medusae to polyp must involve a substitution of cell types and switch into new cell lineages. Consequently, the ontogeny reversal of *T. dohrnii* involves cell transdifferentiation, a reprogramming of structural and functional commitment and gene expression of well-differentiated, somatic cells into other cell types either directly, or through a preparatory return to a state of poor differentiation (Okada 1991; Schmid 1992).

The process of cell transdifferentiation is distinct from cell dedifferentiation. In dedifferentiation, cells that are terminally differentiated (*i.e.*, have lost the ability for proliferation (Sacco *et al.* 2002)) regain the capacity to divide by regressing back (*i.e.*, de-differentiating) into a less-specialized state within the same lineage (Jopling *et al.* 2011; Sugimoto *et al.* 2011; Eguizabal *et al.* 2013). On the other hand, cells that undergo transdifferentiation are multipotent and can switch to any cell lineage. Though transdifferentiation has been reported in *T. dohrnii*, the process of de-differentiation cannot be ruled out and may occur simultaneously with transdifferentiation during regenerative processes. Transdifferentiation has also been reported in other hydrozoans, such as *Hydra vulgaris* (Bode *et al.* 1986; Siebert *et al.* 2008; Galliot and Chera 2010) and *Podocoryna carnea* (Schmid *et al.* 1982; Seipel *et al.* 2004; Schmid and Alder 1984), that have exaggerated longevity or the ability to undergo life-cycle reversal. Overall, the medusa-to-polyp transformation in *T. dohrnii* displays dynamic ontogenetic events, in which a combination of transdifferentiation, asymmetric cell division and apoptotic events occur to rejuvenate into an earlier life-cycle stage.

The cyst stage represents an intermediate step between a regressing, aging, or damaged medusa and a new, healthy polyp. It is also the stage in which tissue reorganization and cell transdifferentiation – starting from the regressing medusa – proceed further and gradually prepare the re-expression of the polyp phenotype. Investigating the gene expression patterns of the cyst is thus interesting from an organismal, developmental, cellular, and molecular perspective. In this paper we present a comparative analysis of the gene expression landscapes across the different life stages of *T. dohrnii*. More specifically, we produced: 1) the first transcriptome assembly and annotation of three stages in the medusa-to-polyp life cycle reversal, *i.e.*, the medusa, cyst and polyp stages; 2) comparative GO annotation and functional gene enrichment analyses to identify GO terms, with a focus on biological processes that are over-or under-expressed in the cyst stage as compared to the polyp and medusa stages. This work provides baseline information to understand the molecular mechanisms underlying both cell transdifferentiation and life cycle reversal in *T. dohrnii*.

## Methods

### Specimen collection, rearing and identification

*T. dohrnii* colonies bearing medusa buds were collected in Otranto, Italy in July 2013. Individual colonies were kept isolated in glass bowls to collect newly released medusae. The colony and medusae were preserved separately in RNAlater RNA stabilization solution (ThermoFischer Scientific) and stored in -80C for further processing. In Bocas del Toro, Panama in July 2015, newly released medusae from polyps found in the field were isolated in glass bowls and starved to induce into cysts (24-48 hr). Cysts were preserved in RNAlater and stored in -80C for further processing. Some polyp tissue was preserved for DNA barcoding purposes. Prior to RNA extraction, total DNA was extracted from polyp tissue from both collections using a protocol provided by Miglietta and Lessios (2009) (Miglietta and Lessios 2009). A fragment of the mitochondrial 16S gene was amplified and sequenced using forward SHA and reverse SHB primers [Forward (SHA): 5′-TCGACTGTTTACCAAAAACATAGC-3′, Reverse (SHB): 5′-ACGGAATGAACTCAAATCATGTAAG-3′] for DNA barcoding to ensure species identification (see (Miglietta and Lessios 2009; Miglietta *et al.* 2018; Moura *et al.* 2011; Schuchert 2014)). The 16S sequences confirming species identification have been deposited in GenBank under the accession number KT984715 for the polyp/medusa and MH029858 for the cyst.

### RNA extraction and cDNA library construction

Total RNA was extracted from the multiple polyps and a pooled sample of medusae (approximately 25-50 individuals for each stage) collected in Italy using Qiagen’s RNAeasy Mini Kit (Cat. #: 74104) following manufacturer’s instruction. Epicentre’s MasterPureTM RNA Purification Kit (Cat. #: MCR85102) was used to extract total RNA from multiple replicates of individual cysts collected in Panama. The protocol was carried out with the following minor modifications recommended by the manufacturer: 1) Pre-rinsing and removing RNAlater from tissue using the T&C Buffer provided by the Epicentre kit; 2) 1/5^th^ of reagent amounts were utilized due to the small amounts of tissue; 3) Increase the number of ethanol washes to additionally purify RNA. The concentration of RNA extracts were determined using the ThermoScientific NanoDrop2000 Spectrometer, and their integrity was validated through the Agilent Technologies Bioanalyzer prior to initiating library construction for sequencing (Additional File A). Three replicates of the cyst stage with the highest quantity/concentration (ng/uL) of DNA based on NanoDrop readings, and highest quality of RNA based on the detection of the strongest 18S (1.8kb-2kb) and 28S (4kb-5kb) bands with the least degradation, samples C1, C2 and C3, were selected for further processing.

### RNA-sequencing and transcriptome assembly

The sequencing library for the polyp stage was constructed by University of Notre Dame’s Genomic and Bioinformatic Core Facility using the TruSeq RNA Sample Prep Kit v2 (Illumina Sequencing Technologies (Cat. #. RS-122-2001)) following manufacturer’s protocols. The construction of the medusa and cyst library was conducted at TAMU’s Agrilife Genomic and Bioinformatics sequencing facility. Due to the small amount of output RNA (less than 100 ng), a pre-prep amplification step using a Poly-A based SMARTer Ultra Low Input RNA for Sequencing v4 kit (Clontech Laboratories (Cat .#: 634888)) was performed prior to library preparation according to manufacturer’s protocol. The cDNA from the polyp library was sequenced using the Illumina MiSeq platform (Illumina Sequencing Technologies) generating 83bp paired-end (PE) reads (Additional file B). The cDNA from the medusa library was sequenced using the Illumina HiSeq2500v4 platform generating 125bp PE reads. The cDNA from the cyst libraries were individually sequenced using the Illumina HiSeq4000 platform generating 150bp PE reads for each library. The raw RNA-seq datasets have been deposited into GenBank under NCBI BioProject ID: PRJNA563171; Accession #: SAMN12669943 - SAMN12669945.

All transcriptomes were constructed individually with the consistent assembly methods. Though acknowledged as a non-traditional approach to perform comparative transcriptomic analyses with individually constructed datasets, due the difficulty in collecting all three stages of *T. dohrnii*, the stages were collected and data processing was performed at different times and localities. RNA-seq reads from the polyp and medusa were processed through the Trinity version 2.2.0 software pipeline where quality trimming via trimommatic (MacManes 2014), read normalization via *in silico normalization*, and *de novo* transcript assembly was conducted using default parameters (Additional file B). The three libraries from the cyst were aligned separately to the assembled polyp and medusa assembly to briefly assess quality of reads. Two of the datasets that had high alignment percentages and low percentages of broken reads were chosen (Additional File C) and pooled together. The pooled reads were processed using the Trinity pipeline using default parameters for transcriptome assembly. Assembly statistics were generated using the trinitystats.pl script within Trinity’s toolkit.

### Quality assessments

Normalized reads (resulting from Trinity’s *In silico normalization*) from all three stages were mapped back to the assembled contigs via TopHat2.1 and CLC Genomic Workbench v8 alignment software using default parameters to evaluate the quality of the assemblies (Additional File D). The BUSCO v2.0 software (Simão *et al.* 2015) was used to assess the completeness of gene content in each assembly. The Eukaryota and Metazoa database provided by the software website (http://busco.ezlab.org) was used for the assessment with default settings (e-value of e^-3^).

### Functional gene annotation

The annotation pipeline from Blast2GO (Conesa *et al.* 2005; Götz *et al.* 2008) was used to assigned functional annotation to the three stage-specific transcriptomes. BLASTx was performed through CloudBlast (Matsunaga *et al.* 2008) against NCBI’s Non-Redundant (NR) database using the following parameters: e-value of e^-3^, word size of 6, HSP length cutoff of 33, and top 20 hits were saved. Gene Ontology (GO) functional annotation was conducted using the following parameters: annotation cutoff of 55, GO weight of 5, expectation value of e^-6^ and HSP-Hit coverage cutoff of 0. InterProScan (Zdobnov and Apweiler 2001) was also used to perform protein domain-based searches against the following databases: BlastProDom, FPrintScan, HMMPIR, HMMPfam, HMMSmart, HMMTigr, ProfileScan, HAMAP, SuperFamily, HMMPanther, Gene3Ds. ANNEX Augmentation was performed after merging the InterPro annotations, and 1^st^ level annotations were manually removed as recommend by B2G (Conesa and Götz 2008). Additionally, the Kyoto Encyclopedia of Genes and Genomes (KEGG) database was used to retrieve enzyme codes and further annotate our transcriptome (Additional File E).

Due to the predicted ecological contamination in the polyp transcriptome of taxa closely related to the Protista species *Reticulomyxa filosa* (Additional File G), sequences that had top hits to *R. filosa* were re-blasted against the metazoan database and the annotation pipeline was re-performed.

### Comparative functional gene enrichment analyses

Functional gene enrichment analyses were conducted using the FatiGO (Al-Shahrour *et al.* 2004) package within the data mining tools in B2G. Prior to the analyses, all three datasets of the stages were combined into one dataset and a sequence ID list that corresponds to each stage’s dataset was generated. Fischer’s Exact Test (two-tailed) was performed using the False Discovery Rate (FDR) p-value of 0.01 to compare the datasets of the following: 1) Cyst *vs.* Polyp; 2) Cyst *vs.* Medusa; 3) Cyst *vs.* Polyp+Medusa combined. The biological processes domain was chosen for the enrichment analyses.

### Data availability

All transcriptome assemblies and annotations are available through request to the main authors. All supplementary files have been uploaded to figshare. The supplementary additional files contain the following: A) NanoDrop and Bioanalyzer results to assess quality; B) Number of raw, trimmed and normalized reads for each stage; C) Cyst RNA-seq alignment to evaluate triplicate datasets; D) Read mapping evaluation of each transcriptome; E) KEGG enzyme mapping; F) De novo assembly statistics, gene content completeness, and functional annotation comparison among cnidarian transcriptomes; G) BLASTx top hit species distribution; H) Summary of Blast2GO transcriptome annotation; I) Names of transcripts from BLASTx annotation and corresponding stages they were identified in; J) Cyst vs. Polyp GO comparative analysis; K) Cyst vs. Medusa GO comparative analysis; L) Cyst vs. Polyp+Medusa GO comparative analysis; M) Top 10 over-expressed biological processes categories in the Cyst as compared to the Polyp and Medusa; N) Annotation profiling of the life cycle stages; O) Ranking of top 100 GO terms based on direct GO count for the Cyst, Polyp and Medusa; P) Reciprocal tBLASTx search of telomerase related mRNA transcripts; Q) Top 10 under-expressed biological processes categories in the Cyst as compared to the Polyp; R) GO categories associated with cellular communication and signaling, cell differentiation and specialization, and development that were under-expressed in the Cyst in comparison to the Polyp and the Medusa. The RNA-sequencing datasets generated and analyzed during the current study are available in NCBI’s GenBank’s repository under NCBI BioProject ID: PRJNA563171, Accession #: SAMN12669943 - SAMN12669945. Supplemental material available at figshare: https://doi.org/10.25387/g3.9943517.

## Results and discussion

### Transcriptome assembly and quality assessments

Polyps and newborn medusae were collected in Otranto, Italy. Cysts were induced and collected in Bocas del Toro, Panama. All specimens were barcoded using the mitochondrial 16S gene to confirm identification. The 16S sequence showed 100% similarity and identity with *T. dohrnii* (GenBank accession#: KT984715 for Polyp and Medusa, MH029858 for Cyst; also see Miglietta *et al.* 2018 (Miglietta *et al.* 2018) for a phylogeny of the genus *Turritopsis*).

Although chosen polyps were not actively budding medusae, we cannot exclude the presence of developing medusa buds. RNA from pooled polyps (*i.e.*, multiple polyps in colony) were extracted, prepped into cDNA libraries and sequenced using the Illumina MiSeq platform (83bp PE reads) and the medusa using HiSeq2500v4 (125bp PE reads). RNA was extracted from individual cysts and individual libraries were sequenced using HiSeq4000 (150bp PE reads). Due to the different approaches in number of individuals used for extraction (*i.e.*, pooled *vs.* individual), different times and locality of specimen collected and rearing laboratory, library preparation and sequencing, the sequences from the cDNA libraries were assembled separately into three individual, stage-specific transcriptomes (Additional File B).

From the stage-specific RNA-seq libraries, 92,659, 74,639 and 86,373 contigs were assembled *de novo* for the Polyp, Medusa, and Cyst stages respectively using the Trinity assembler (Haas *et al.* 2013) (Table 1). The assembled transcriptomes had a N50 value of 1,332bps for the Polyp stage, 1,434 bps for the Medusa, and 1,634bps for the Cyst. We compared our transcriptomes with those of other published Cnidaria belonging to five classes: Hydrozoa, Scyphozoa, Cubozoa, Anthozoa and Myxozoa (Atkinson *et al.*, 2018). Although there were differences in sequencing, assembly and annotation pipelines, our transcriptomes have comparable assembly statistics, completeness and annotated sequences as other published cnidarian transcriptomes (Hasegawa *et al.* 2016; Wenger and Galliot 2013; Sanders *et al.* 2014; Sanders and Cartwright 2015a, 2015b; Ortiz-González *et al.* 2017; Hroudova *et al.* 2012; Brekhman *et al.* 2015; Ponce *et al.* 2016; Li *et al.* 2014; Brinkman *et al.* 2015; Ames *et al.* 2016; Magie *et al.* 2005; Kitchen *et al.* 2015; Ayala-Sumuano *et al.* 2017; Sunagawa *et al.* 2009; Mehr *et al.* 2013; Huang *et al.* 2016; Chang *et al.* 2015) (Additional File F).

**Table 1:** Trinity *de novo* assembly statistics. The trinitystats.pl script from the Trinity toolkit utilities was used to generate assembly statistics based on overall transcript length for all three transcriptomes

	Polyps	Medusa	Cyst
**# of unique transcripts**	76,456	58,312	62,666
**# of transcripts**	92,659	74,639	86,373
**% GC**	37.33	37.17	38.25
**Contig N10**	3,576	3,766	3,935
**Contig N30**	2,116	2,196	2,420
**Contig N50**	1,332	1,434	1,634
**Median contig length**	376	493	645
**Mean contig length**	737.35	864.82	1,010.57
**Minimum length**	201	201	201
**Maximum length**	11,079	20,570	20,736
**Total assembled bases**	68,322,250	64,550,812	87,289,185

Normalized reads resulting from Trinity’s *in silico normalization* were mapped back to the assembled contigs for each transcriptome to evaluate the completeness of its assembly. Two different alignment software based on different algorithms (TopHat v2.1 and CLC Genomic Workbench v8 read mapper tool) were utilized for the assessment. In both assessments, the Polyp assembly exhibited more than 10% lower mapping percentages (TopHat: 69.5%, CLC: 84.2%) than the Medusa (81.2%, 95.1%) and Cyst (80.0%, 98.8%) (Additional File D).

It is acknowledged that different library preparation and sequencing methods (*e.g.*, platform, read length, depth, etc.) could affect the overall length and contiguity of the assembled contigs. This was reflected in our assembly statistics (Table 1), as the Polyp transcriptome, constructed from 83bp PE Illumina MiSeq reads, had the shortest contigs among the three transcriptomes. The Cyst stage on the other hand, assembled from the longest150 bp PE Illumina HiSeq reads, had the longest contigs. Thus, the completeness of our transcriptome was further tested using the BUSCO v2.0 software (Simão *et al.* 2015), which provides insight on whether a significant amount of genetic information was lost in unmapped reads, and on whether the sequencing method had significant impacts on the completeness of the transcriptome in terms of gene content and transcript fragmentation. Using BUSCO v2.0, we conducted assessments on each stage-specific assembly using the Metazoa database. All assemblies exhibited high coverage and completeness (above 90%) in terms of gene content (Table 2). The Polyp stage had the highest percentage of complete BUSCOs (95.4%), the Medusa had the second highest (93.0%), and the Cyst stage had the lowest percentage (91.3%). This implies that despite exhibiting the lowest percentage of reads mapped back to the contigs (Additional File D), no significant amount of genetic information was lost in the unmapped reads. In addition, though the Polyp transcriptome was assembled using shorter sequencing reads and less depth than the Medusa (125bp PE reads via HiSeq2500v4) and Cyst (150bp PE reads via HiSeq4000), the number of fragmented BUSCOs were lower than the other stages, 2.2% as opposed to 3.4% and 3.6%, respectively (Table 2). Overall, our assessments suggest that despite the differences in library preparation and sequencing approaches, all three transcriptomes have high coverage and will provide quality foundations for further analyses.

**Table 2:** BUSCO transcriptome quality assessment. The BUSCO analyses were conducted using the Metazoa database

	Metazoa database (Toal BUSCOS: 978)					
	Polyp		Medusa		Cyst	
	#	%	#	%	#	%
**Complete BUSCOs**	933	95.4%	909	93.0%	893	91.3%
**Fragmented BUSCOs**	22	2.2%	33	3.4%	35	3.6%
**Missing BUSCOs**	23	2.4%	36	3.6%	50	5.1%

### Transcriptome annotation and characterization

The Blast2GO pipeline was used to assign names and functional terms to the assembled contigs of each transcriptome. InterProScan (Zdobnov and Apweiler 2001) was also used to perform protein domain-based searches using InterPro databases (refer to methods for databases) to build upon and confirm existing GO annotations. Enzyme mapping against the KEGG database was also performed for each stage (Additional File E) and EC numbers were added to the annotations. An expectation value cutoff of e^-3^ was utilized to perform BLASTx against the NR database. An expectation value cutoff of e^-3^ is generally considered an acceptable but liberal threshold (De Wit *et al.*, 2012). This threshold was chosen as our work is exploratory as there were no reported transcript annotations or genome for any *Turritopsis* species. We also did not want to limit our search to the few published hydrozoans, which have not been reported to undergo reverse development and do not share similar life histories (*e.g.*, medusa stage is absent, does not form colonies).

There was a concern of contamination of epibiontic organisms in the Polyp transcriptome, specifically the foraminiferan *Reticulomyxa filosa* (Protista), which showed up in the top-blast hits (Additional file G). Sequences with top-hits to *R. filosa* were re-blasted using the Metazoa database to best filter out sequences and annotations that originated from protists.

**Figure 2:**
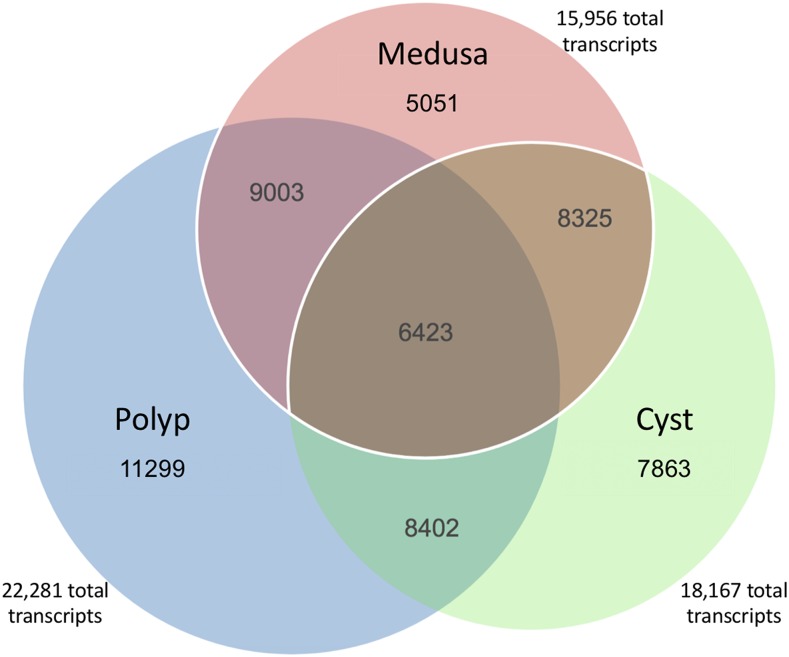
Venn diagram of the Blastx annotations from the Polyp, Medusa and Cyst stage. Represents the number of shared and stage-exclusive transcripts (*i.e.*, Blastx descriptions).

45,405 out of 92,659 (49.00%) contigs in the Polyp, 33,836 out of 74,639 (45.33%) contigs in the Medusa, 38,565 out of 86,373 (44.65%) contigs in the Cyst assembly showed significant similarity to proteins in the NR database in the BLASTx step (Additional File H). The majority of the top-hits belonged to four cnidarian species, *H. vulgaris*, *E. pallida*, *A. digitifera*, and *N. vectensis*, ranked 1^st^ to 4^th^ in the same order in all three stages (Additional File G, B-D). This indicates that all three transcriptomes are highly composed of transcript annotations from our target organism (*i.e.*, *T. dohrnii*) as opposed to contaminant, non-target organisms that may naturally occur with *T. dohrnii* (*e.g.*, protozoans, fungi, etc.). Ultimately, there were 38,686, 27,443, and 31,633 contigs annotated with at least one GO term in the Polyp, Medusa and Cyst, respectively (Table 3). In all, the BLASTx annotation identified 11,299, 5,051 and 7,863 Polyp, Medusa and Cyst-specific transcripts, respectively (Figure 2, names of transcripts found in Additional File I). The Cyst shared 8,402 transcripts with the Polyp and 8,325 with the Medusa, and 6,423 with both stages. There were 9,003 transcripts that were not found in the Cyst stage.

**Table 3:** Number of GO categories in biological processes that were over- or under-expressed in each comparative test. Three separate comparisons were performed: Cyst *vs.* Polyp, Cyst *vs.* Medusa, and Cyst *vs.* Polyp+Medusa. An FDR adjusted p-value (q-value) of 0.01 was utilized in all three analyses

		Cyst *vs.* Polyp	Cyst *vs.* Medusa	Cyst *vs.* Polyp+Medusa
Biological processes	Over	44 (3.27%)	147 (64.19%)	66 (8.99%)
	Under	1302 (96.73%)	82 (35.81%)	734 (91.01%)
	Total	1346	229	780

Our BLASTx results differed from a recently published transcriptome of various stages of an undetermined *Turritopsis* species (Hasegawa *et al.* 2016), where *H. vulgaris* and *N. vectensis* ranked 1^st^ and 2^nd^ among top hit species, and accounted for 38% of all top-hits. Our *T. dohrnii* transcriptomes incorporate 43.88–57.44% of top-hits with *H. vulgaris* alone, and between 56.88% and 73.77% when top-hits from the highest ranked cnidarian species are combined (Additional File G, B-D). In addition, the published Hasegawa *et al.* (2016)(Hasegawa *et al.* 2016) transcriptome shows a gram-negative bacterium species (*Acidovorax* sp. KK102) as the 3^rd^ highest top-hit species, which is most likely the outcome of contamination.

### Comparative functional gene enrichment analyses

Categorizing the data based on GO terms creates the opportunity to understand specific physiological components found in different *T. dohrnii* stages, and to assess the functional differences among them. We performed comparative functional gene enrichment analyses on the Cyst, Polyp and Medusa transcriptomes, with the Cyst as the central stage of comparison, to gain more insight on the over and under-expressed GO categories in the Cyst. We acknowledge that our transcriptomes assemblies are catalogs of transcripts and thus, there is no separation between lowly expressed and highly expressed transcripts, and therefore cannot detect differential expression of specific transcripts. Our enrichment results are preliminary and need further confirmation with normalized library replicates for each stage. However, these comparative analyses provide useful and novel insights on the processes that are active and suppressed in the Cyst.

We focused on the biological processes domain that includes recognized series of molecular events and networks pertinent to the functionality of the cell, organs, or organism as a whole. The FatiGO (Al-Shahrour *et al.* 2004) tool statistically assesses differences in functional annotation between datasets using the Fischer’s Exact Test (two-tailed) and a stringent False Discovery Rate (FDR) adjusted p-value (q-value) of 0.01. We show the results from the following comparisons: 1) Cyst *vs.* Polyp; 2) Cyst *vs.* Medusa; and 3) Cyst *vs.* Polyp+Medusa. Due to the larger number of total contigs with GO annotations in the Polyp transcriptome, the Cyst *vs.* Polyp+Medusa will show slight bias toward the Polyp stage in the reference dataset. This test however was designed to uncover the over- and under-expression of gene categories that are highly specific to the Cyst.

#### GO enrichment analyses:

The comparative gene enrichment analyses revealed biological pathways associated with GO terms that were over- and under-expressed in each of the comparative tests (Table 3). In total, there were more under-expressed GO categories than over-expressed in the Cyst as compared to the Polyp and Medusa stage. However, in the Cyst *vs.* Medusa analysis, there were more over-expressed GO categories than under-expressed. This indicates that though the cyst is seemingly dormant, there is much more cellular activity occurring than previously envisioned.

Due to the large number of reported categories, we reduced the output of the Cyst *vs.* Polyp and Cyst *vs.* Medusa comparisons to the most specific GO term, and visualized the top 50 most comparatively enriched categories in an enriched bar graph (Figure 2).

The top three most specific GO categories significantly over-expressed in the Cyst when compared to the Polyp were related to DNA biosynthesis, integration, and telomere maintenance (reference to GO:0006278, GO:0015074, GO:0000723) (Figure 2A). The top three most specific GO categories enriched in the Cyst when compared to the Medusa (Figure 2B) were similar to what was reported in the Cyst *vs.* Polyp comparison (Figure 2A), with the discrepancy of ‘DNA repair (GO:0006281)’ being present instead of ‘Telomere maintenance’. Telomere maintenance is typically portrayed as a type of DNA repair mechanism and thus, our results indicate that DNA repair processes (*e.g.*, DNA repair, telomere maintenance), along with DNA integration, and DNA biosynthesis related processes, may have a significant role in the regenerative events occurring in the Cyst.

**Figure 3:**
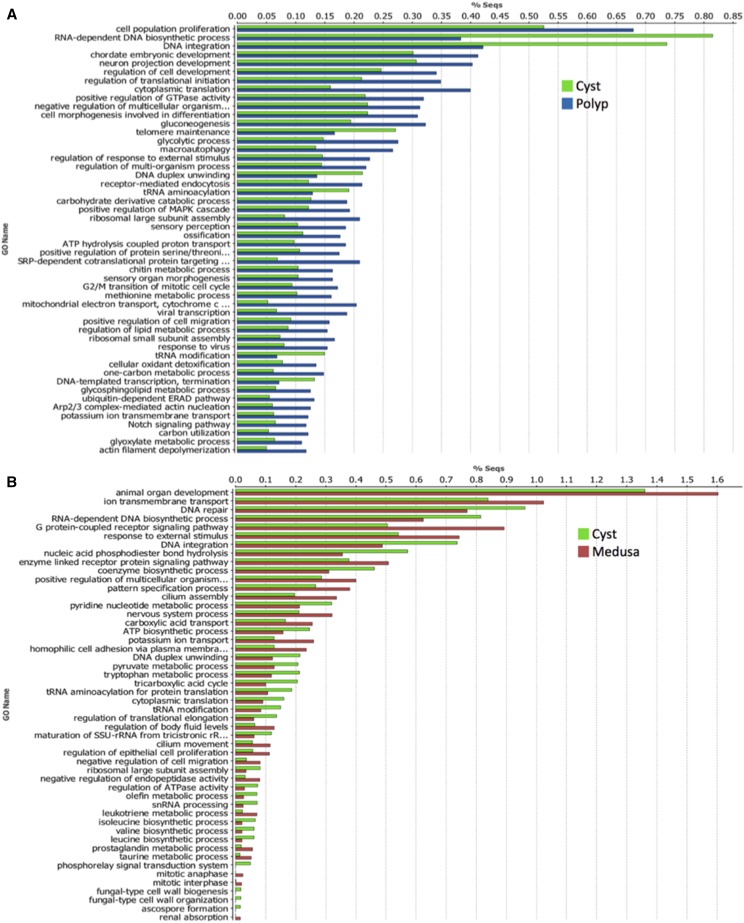
A) Cyst *vs.* Polyp comparative functional gene enrichment analyses reduced to the most specific biological processes categories. B) Cyst *vs.* Medusa comparative functional gene enrichment analyses reduced to the most specific biological processes categories.

On the other hand, significantly enriched GO terms in the Polyp were associated with cellular proliferation and embryonic and neural development (reference to GO:0008283, GO:0043009, GO:0031175) (Figure 2A). ‘Chordate embryonic development’ was an unusual GO category to find enriched in any stage of *T. dohrnii*, an invertebrate, basal metazoan. Such annotated transcripts likely represent ancestral orthologous transcripts involved in embryonic development that have been conserved throughout lower and higher metazoans, similarly reported with bilaterian specific genes found in the hydrozoan *Clytia hemisphaerica* (Leclère *et al.* 2019), and ancestral non-metazoan ESTs have been conserved and found in *Nematostella vectensis* and *Acropora millepora* (Technau *et al.* 2005). The most specific processes enriched in the Medusa when compared to the Cyst were associated to organ development, transport and signaling pathways (reference to GO:0048513, GO:0034220, GO:0038042). Medusae of *Turritopsis* sp. have also been reported to highly express genes associated to cnidarian axial patterning and development, such as the Wnt pathway (Hasegawa *et al.* 2016). Though the same was not reported for the Polyp stage, our analyses indicate that both stages incorporate more signaling (MAPK, Notch, etc.), morphogenesis and development categories than the Cyst stage (Figure 3).

Overall, DNA integration, repair and telomere maintenance related processes were highly enriched in the Cyst stage as compared to the Polyp and Medusa. System and cellular development, transport and signaling pathways were found to be suppressed in the Cyst as compared to the Polyp and Medusa. Here below, we further analyze specific GO categories and their child terms that are of interest for further exploration (Additional Files J, K, L).

### Over-expression in the Cyst

The top 10 over-expressed gene enrichment categories for each analysis are cumulatively reported in Additional File M. In the Cyst *vs.* Polyp comparison, most of the highest expressed categories are directly involved in DNA synthesis, integration, and metabolic processes. The top 10 over-expressed gene enrichment categories in the Cyst *vs.* Medusa comparison involved processes such as the synthesis and metabolism of cellular compounds, particularly related to nitrogen. Cellular metabolic processes involving nucleic acid constituents (in reference to GO:006139) were both found to be highly enriched in the Cyst in comparison to the Polyp (ranked 7^th^) and Medusa (ranked 8^th^). When the Cyst was compared to the Polyp and Medusa combined, DNA metabolic processes were the top over-expressed categories.

#### DNA integration, transposition, and repair:

‘DNA integration (GO:0015074)’, was among the top 10 over-expressed categories in the Cyst *vs.* Polyp and Cyst *vs.* Polyp + Medusa analyses (Additional File L). Moreover, the Cyst *vs.* Medusa comparison indicated that there was an enrichment of ‘DNA integration’ in the Cyst (Table 4). The Cyst incorporated 636 transcripts with the DNA integration annotation, while the Polyp and Medusa incorporated a substantially smaller number of sequences in the category, 390 and 364 transcripts, respectively.

**Table 4:** GO categories significantly over- and under-expressed in the Cyst. A) DNA integration, transposition and repair, B) Telomere regulation, C) Lifespan and aging, D) Cell division, E) Response to stimuli

Cat.	Exp.	GO ID	GO Name	Analysis	q-value	p-value	#GO Test	#GO Ref
A) DNA integration, transposition and repair	Over	GO:0015074	DNA integration	Cyst *vs.* Polyp	5.73e^-17	8.31e^-19	636	390
				Cyst *vs.* Medusa	4.72e^-08	1.78e^-10	636	364
				Cyst *vs.* Polyp+Medusa	3.78e^-17	1.56e^-19	636	754
		GO:0032196	Transposition	Cyst *vs.* Polyp	2.73e^-03	2.55e^-04	76	40
				Cyst *vs.* Polyp+Medusa	3.35e^-03	1.80e^-04	76	80
		GO:0032197	Transposition, RNA-mediated	Cyst *vs.* Polyp	2.73e^-03	2.55e^-04	17	2
				Cyst *vs.* Polyp+Medusa	9.82e^-04	4.23e^-05	17	3
		GO:0006281	DNA repair	Cyst *vs.* Polyp	2.76e^-03	2.62e^-04	831	742
				Cyst *vs.* Medusa	2.27e^-03	3.45e^-05	831	574
				Cyst *vs.* Polyp+Medusa	1.74e^-04	5.88e^-06	831	1316
B) Telomere regulation	Over	GO:0000723	Telomere maintenance	Cyst *vs.* Polyp	3.50e^-05	2.04e^-06	234	154
				Cyst *vs.* Polyp+Medusa	4.12e^-04	1.60e^-05	234	310
		GO:0032200	Telomere organization	Cyst *vs.* Polyp	1.06e^-04	6.86e^-06	236	160
				Cyst *vs.* Polyp+Medusa	1.49e^-03	6.25e^-05	236	323
C) Lifespan and aging	Under	GO:0008340	Determination of adult lifespan	Cyst *vs.* Polyp	2.46e^-13	4.81e^-15	7	77
				Cyst *vs.* Polyp+Medusa	1.27e^-08	1.44e^-10	7	94
		GO:0010259	Multicellular organism aging	Cyst *vs.* Polyp	4.60e^-15	7.50e^-17	7	84
		GO:0007568	Aging	Cyst *vs.* Polyp	7.90e^-07	3.39e^-08	70	161
D) Cell Division	Under	GO:0098763	Mitotic cell cycle phase	Cyst *vs.* Polyp	3.01e^-11	7.19e^-13	2	52
				Cyst *vs.* Medusa	2.37e^-07	1.03e^-09	2	34
				Cyst *vs.* Polyp+Medusa	3.68e^-11	2.80e^-13	2	86
		GO:0000087	Mitotic M phase	Cyst *vs.* Polyp	4.17e^-10	1.14e^-11	1	43
				Cyst *vs.* Medusa	9.75e^-06	6.46e^-08	1	25
				Cyst *vs.* Polyp+Medusa	1.65e^-09	1.50e^-11	1	68
		GO:0000090	Mitotic anaphase	Cyst *vs.* Polyp	1.82e^-09	5.42e^-11	0	36
				Cyst *vs.* Medusa	1.06e^-04	9.76e^-07	0	18
				Cyst *vs.* Polyp+Medusa	1.95e^-08	2.32e^-10	0	54
		GO:0051329	Mitotic interphase	Cyst *vs.* Medusa	6.92e^-03	1.36e^-06	1	15
				Cyst *vs.* Polyp+Medusa	3.69e^-03	1.94e^-04	1	27
		GO:0007067	Mitotic nuclear division	Cyst *vs.* Polyp	1.28e^-07	5.02e^-09	200	357
				Cyst *vs.* Polyp+Medusa	3.19e^-04	1.49e^-05	200	550
		GO:0007052	Mitotic spindle organization	Cyst *vs.* Polyp	5.88e^-06	2.98e^-07	49	121
				Cyst *vs.* Polyp+Medusa	1.61e^-04	7.39e^-05	49	176
		GO:2000045	Regulation of G1/S transition of the mitotic cell cycle	Cyst *vs.* Polyp	7.98e^-05	5.07e^-06	44	105
				Cyst *vs.* Polyp+Medusa	9.80e^-03	6.30e^-04	44	151
		GO:0000080	Mitotic G1 phase	Cyst *vs.* Polyp+Medusa	7.65e^-03	4.61e^-04	0	20
F) Response to stimuli	Over	GO:0033554	Cellular response to stress	Cyst *vs.* Medusa	9.64^-03	2.00e^-04	1434	1067
	Over	GO:0006974	Cellular response to DNA damage stimulus	Cyst *vs.* Polyp+Medusa	8.60e^-03	5.36e^-04	999	1684
	Under	GO:0050896	Response to stimulus	Cyst *vs.* Polyp	4.47e^-47	8.05e^-50	5005	6992
				Cyst *vs.* Medusa	2.34e^-04	2.42e^-06	5005	4745
				Cyst *vs.* Polyp+Medusa	1.95e^-29	2.25e^-32	5005	11737
		GO:0009628	Response to abiotic stimulus	Cyst *vs.* Polyp	8.71e^-24	7.10e^-26	315	676
				Cyst *vs.* Polyp+Medusa	7.05e^-13	4.55e^-15	315	994
		GO:0009607	Response to biotic stimulus	Cyst *vs.* Polyp	1.12e^-15	1.75e^-17	167	383
				Cyst *vs.* Polyp+Medusa	4.91e^10	4.30e^-12	167	578

In addition, the GO categories related to transposition (reference to GO:0032196, GO:0032197) were over-expressed in the Cyst *vs.* Polyp and Cyst *vs.* Polyp+Medusa analyses (Table 4). The Cyst incorporated 76 transcripts in the ‘Transposition’ category and 17 transcripts in the ‘Transposition, RNA-mediated’ category. On the other hand, the Polyp and Medusa incorporated only 40 transcripts in the ‘Transposition’ category and 2 and 1, respectively in the ‘Transposition, RNA-mediated’ category. It was noted that the Polyp and Medusa incorporated similar numbers of transcripts in each of the categories.

Furthermore, in the Cyst stage, the category ‘DNA repair (GO:0006281)’ was also found to be over-expressed in all three comparative analyses (Table 4). The Cyst incorporated 831 transcripts in the DNA repair category, while the Polyp and Medusa incorporated 742 and 574 transcripts in the category, respectively. When the top 20 most enriched GO terms for specific GO levels were explored, DNA repair (GO level 6) was present in the Cyst (rank 18^th^) but absent in the Polyp and Medusa among the 20 terms (Additional Files N). Overall, DNA Repair ranked 14^th^ in the Cyst, 31^st^ in the Polyp and 23^rd^ in the Medusa in GO terms in biological processes for all levels (Additional File O). These results potentially indicate that the Cyst is prioritizing DNA repair processes more than the Polyp and the Medusa.

The combination of the over-expression of DNA integration, transposition, and repair categories in the Cyst indicate that regulation of genome integrity may have an important role in *T. dohrnii*’s reverse development. Active transposons are mutagenic and it has been shown that in *Hydra*, another Cnidarian taxa with regeneration capabilities, transposon repression and regulation by Piwi pathway occurs both in the I-cells and in the somatic cells (Juliano *et al.* 2014; Lim *et al.* 2014). Future research on *T. dohrnii* will focus on regulation of transposable elements and their role in preserving genome integrity in the cells that compose the cyst stage.

#### Telomere maintenance and organization:

Telomeres are protective sequences at the end of chromosomes that shortened each time the cell undergoes DNA replication, and this loss of genetic material is attributed to cellular senescence and the aging phenotype. In immortal cell lineages, such as germ cells and cancerous cells, telomere length is maintained by the action of the telomerase enzyme (Kelland 2007; Low and Tergaonkar 2013). The categories ‘Telomere maintenance (GO:0000723)’ and ‘Telomere organization (GO:0032200)’ was over-expressed in the Cyst as compared to the Polyp and both stages combined. The Cyst assembly resulted in 234 and 236 contigs respectively in the two categories, while the Polyp possessed only 154 and 160 contigs (Table 4). Our results also suggest that the Medusa incorporated fewer number of contigs pertaining to telomere maintenance and organization, 156 and 163, respectively (Additional File K). It was once again noted that the Polyp and Medusa stages exhibit a similar number of contigs associated with the two categories related to telomeric regulation, which significantly increase in the Cyst, during the course of reverse development.

The telomerase reverse transcriptase isoform x1 enzyme was found annotated in all three of transcriptomes with a e-value of 0.00. In addition, genes that associate with telomerase such as ‘telomerase component 1’, ‘telomerase binding EST1A’ ‘PIN2 TERF2-interacting telomerase inhibitor’ and ‘regulator of telomere elongation helicase 1’ were found annotated in our transcriptomes. Furthermore, reciprocal BLAST analyses were performed to determine whether specific components of telomerase and related transcripts were expressed in our transcriptomes. Our analyses indicated that all queried telomerase subunits (TERT, TERC, DKC1, TEP1) were found in all three stages (Additional File P). DKC1, the Dsykerin component, exhibited an e-value of 0.0 and the highest bit score (714.5) in all stages (Additional File P).

Our results indicate that the regulation of telomere length may be a key component of the overall regulatory network enabling *T. dohrnii*’s life cycle reversal, and should therefore be the focus of future studies.

### Under-expression in the Cyst

The top 10 under-expressed enrichment categories for each analysis are cumulatively reported in Additional File Q. When compared to the Polyp, the prominent processes that are suppressed in the Cyst stage are directly related to the maintenance, specifically the synthesis, of proteins (reference to GO:0019538, GO:00064120)’, GO:0042254). In addition, processes involved in the synthesis of cell components were highly suppressed in the Cyst. When compared to the Medusa, processes involved in cell signaling and communication (reference to GO:0007186, GO:0007165) and mitotic cell division (GO:0098763) are down regulated in the Cyst.

#### Cell communication and signaling:

More than 100 different GO categories involved in cellular communication and signaling were found to be under-expressed in the Cyst when compared to the Polyp and Medusa in at least one of the three analyses (Additional File J, K, L). GO categories that were significant in all three analyses (Cyst *vs.* Polyp, Cyst *vs.* Medusa, Cyst *vs.* Polyp+Medusa) and processes of interest that pertained specifically to cell surface receptor signaling were reported in (Additional File R). The only over-expressed signaling GO category was ‘Phosphorelay signal transduction system (GO:0000160)’, and was found to be over-expressed in all analyses (Additional File J, K, L). Our results are consistent with the finding of Hasegawa *et al.* (2016) (Hasegawa *et al.* 2016) on *T*. sp., in which in the Cyst stage incorporated the lowest representation of transcripts belonging to the cell communication and signaling pathways. Processes associated with the Wnt signaling pathway (reference to GO:0035567, GO:0060071) were found to be under-expressed in the Cyst, particularly when compared to the Polyp.

Wnt-signaling is highly conserved in metazoans and has a critical role in the spatial patterning and axis formation during embryogenesis (Holland *et al.* 2013; Logan and Nusse 2004). ‘Notch signaling pathway (GO:0007219)’ was also reported to be significantly under-expressed in the Cyst (Additional File R). In all, the Cyst incorporated 56 transcripts in the Notch signaling category, while the Polyp and Medusa incorporated 109 and 78 transcripts in the category, respectively. Notch signaling is involved in the molecular control of neurogenesis (Bosch *et al.* 2017) and it is closely associated to the Wnt-signaling cascade in cell fate determination and development (Collu *et al.* 2014; Hayward *et al.* 2008). Indeed, the peptidergic nervous system of the *T. dohrnii* medusa appears almost entirely dismantled in the cyst, where few scattered RFamide- and GLWamide- immunoreactive cells are barely or no longer detectable (A. D’Elia, J. Schmich, and S. Piraino, unpublished data). The inhibition of the Notch signaling pathway has been shown to be associated with cell transdifferentiation in specific systems (Vega *et al.* 2014). Additionally, The G-protein coupled receptor signaling pathway (reference to: GO:0007186) includes processes involved in the transduction of signaling into G-protein complexes, which a variety of signals that are fundamental for cellular development and growth (Tuteja 2009). These results indicate that contact mediate signaling and inhibition of communication between cells in the Cyst are additional interesting aspects of *T. dohrnii* life cycle reversal that are worth of further investigation.

#### Lifespan and aging:

Physical deterioration and the decline in cellular functionality and regenerative potential are characteristics associated with the process of aging. Categories associated with the biological aging of organisms were found to be under-expressed in the Cyst, particularly in comparison to the Polyp (Table 4). The category ‘Determination of adult lifespan (GO:0008340)’, constituting an important aspect of aging, was under-expressed in the Cyst *vs.* Polyp analysis and the Cyst *vs.* Polyp+Medusa analysis. 17 contigs were found to be associated with the ‘Determination of adult lifespan’ category in the Medusa (Additional File K), much fewer than in the Polyp (77 contigs), and much more than in the Cyst (7 contigs). Broader categories of aging (reference to GO:0010259, GO:007568), were also found to be under-expressed in the Cyst when compared to the Polyp stage (Table 4).

#### Cell division:

A considerable number of GO categories associated with mitotic cell division were suppressed in the Cyst when compared to the other stages, with 27 total categories under-expressed in the Cyst *vs.* Polyp comparison (Additional File J), 4 under-expressed in the Cyst *vs.* Medusa (Additional File K), and 12 under-expressed in the Cyst *vs.* Polyp and Medusa combined (Additional File L (see Table 10 for the most significant)). ‘Mitotic cell cycle phase (GO:0098763)’ was the 9^th^ overall most under-expressed category in the Cyst when compared to the Medusa (Additional File K).

Despite the fact that gene categories related to mitotic cell division were under-expressed in the Cyst, processes involved in DNA synthesis, maintenance, integration, transposition and repair, were significantly over-expressed (Additional Files R, Table 4) in the Cyst as compared to the other stages. This is particularly interesting as it indicates that though the cells in the Cyst are not dividing, they are highly active in generating, modifying and repairing their genome.

#### Response to stimuli:

The majority of processes associated with the response to external and internal stimuli were significantly under-expressed in the Cyst as compared to the Polyp and Medusa. Specifically, there were 142 under-expressed categories when the Cyst is compared to the Polyp (Additional File J), 3 when compared to the Medusa (Additional File K), and 84 when compared to both stages combined (Additional File L). Due to the large number of under-expressed categories, the results were condensed to represent broader categories, along with the only two exceptions of categories associated with the response to stimuli over-expressed in the Cyst (Table 4), namely ‘Cellular response to stress (GO:0033554)’ and ‘Cellular response to DNA damage stimulus (GO: 0006974)’. The over-expression of these categories likely reflects the nature of the study system in which reverse development is induced by stressors, while the inhibition of a large number of pathways associated to the response to stimuli in the Cyst is likely attributed to the lack of sensory organs, structures, and recognizable nerve cells, and the fact that the ectodermal cells are separated from the outer environment by the perisarc.

#### Cellular differentiation and development:

A large number of categories that are involved in cell differentiation and organization were down-regulated in the Cyst as compared to the Polyp and Medusa stages (see Additional File R for most significant). In addition, 54 categories with the key word ‘development’ were reported to be under-expressed in the Cyst in comparison to the Polyp (Additional File J), 6 categories compared to the Medusa (Additional File K), and 29 categories when compared to both the stages combined (Additional File L). Due to the large number of under-expressed categories associated with organismal development, broader categories were reported in Additional File R.

### Conclusion

RNA-sequencing, *de novo* assembly, and functional annotation were performed to characterize the transcriptome profiles of the Polyp, the Medusa and the Cyst, three life cycle stages of the cnidarian *Turritopsis dohrnii*. Comparative functional gene enrichment analyses were conducted using the Cyst as the central stage of comparison to identify biological pathways that are potentially involved in the reverse development and transdifferentiation in *T. dohrnii*. Our results show that categories associated with DNA synthesis, repair processes, telomerase activity and telomere maintenance are over-expressed in the Cyst when compared to the Medusa and the Polyp stages. On the other hand, transcripts associated to specialized functions involving lifespan and aging, response to stimuli, cell division and cell differentiation and development, are under-expressed in the Cyst when compared to the Medusa and the Polyp stages. Our study of the life-stage specific *T. dohrnii* transcriptomes gives a glimpse of the molecular regulatory gene network controlling the stability and reprogramming of differentiated cells, tissue homeostasis, longevity, and the associated potential for organismal rejuvenation in a promising *in vivo* system. This research has produced a transcriptomic landscape for the analysis of the extraordinary potential of reverse development and cell transdifferentiation in *T. dohrnii*. As a corollary, it suggests the foundation of a new experimental paradigm to gain novel insights on regeneration, cell plasticity, aging, and the directionality of ontogeny in a non-model, *in vivo* metazoan system. Finally, it identifies biological processes such DNA repair and integrity, role of transposable elements, cell to cell communication, and telomerase activity, as processes that occur at the Cyst stage and that are worth of further investigation.
